# Medical Mirror Prize Contest

**Published:** 1900-11

**Authors:** 

**Affiliations:** St. Louis, Mo.


### Correspondence.

### MEDICAL MIRROR PRIZE CONTEST.

St. Louis, Mo., October 11, 1900.

*To the Editor Buffalo Medical Journal:*

*Sir.*—Thanking you for whatever attention you may have shown our previous request in regard to the announcement of our tuberculosis contest, we shall appreciate very much your attention to the a change of date of closing. At the request of many prospective contestants it has been decided to change the date of closure of entries to at least November 15th. This does not mean that the final date for receiving entries has been changed, for it still remains February 1, 1901, the prizes to be distributed by the committee on awards on March 1, suceeding. We feel that our effort is one of unusual liberality and any editorial comment you may make upon it will be appreciated.

THE MEDICAL MIRROR.

